# Clinical Relevance of Plasma Prolylcarboxypeptidase Level in Patients with Idiopathic Acute Optic Neuritis

**DOI:** 10.3390/jcm13072038

**Published:** 2024-04-01

**Authors:** Jong-Heon Kim, Dae Beom Shin, Kyoungho Suk, Bo Young Chun

**Affiliations:** 1 Brain Science & Engineering Institute, School of Medicine, Kyungpook National University, Daegu 41944, Republic of Korea; jongheonkim.phd@gmail.com (J.-H.K.); ksuk@knu.ac.kr (K.S.); 2 Department of Ophthalmology, School of Medicine, Kyungpook National University, Daegu 41944, Republic of Korea; 3 Department of Pharmacology, School of Medicine, Kyungpook National University, Daegu 41944, Republic of Korea

**Keywords:** neuroinflammation, optic neuritis, prolylcarboxypeptidase (PRCP), recurrence

## Abstract

**Objectives:** This study evaluated the plasma concentration of prolylcarboxypeptidase (PRCP) and its clinical relevance in patients with idiopathic acute optic neuritis (ON). **Methods:** We investigated the expression of PRCP in the optic nerves of experimental autoimmune optic neuritis (EAON)-induced mice. Peripheral blood samples were collected from ON patients (n = 20) and healthy controls (n = 20). ELISA was used to measure the plasma PRCP levels. We performed measurements of visual acuity and the mean thicknesses of the macular ganglion cell layer plus inner plexiform layer (GCL+IPL) at diagnosis and 6 months after diagnosis. **Results:** The PRCP mRNA expression in EAON-induced mice was markedly higher than that in naïve mice. The mean plasma PRCP level was significantly higher in patients with ON than in controls. Plasma PRCP levels were negatively correlated with logMAR visual acuity at 6 months after diagnosis and differences in macular GCL+IPL thickness during an ON attack. A plasma PRCP level of 49.98 (pg/mL) predicted the recurrence of ON with a 75% sensitivity and 87.5% specificity. **Conclusions:** Patients with idiopathic acute ON had higher plasma PRCP levels, and this was positively correlated with final visual outcome and well-preserved macular GCL+IPL thickness during an ON attack. The increase in plasma PRCP level may reflect its compensatory secretion to counteract neuroinflammation in ON patients.

## 1. Introduction 

Optic neuritis (ON), an inflammatory demyelinating disorder of the optic nerve, often presents as an acute episode of decreased vision, eyeball pain, and dyschromatopsia in young adults [1]. ON is closely related to multiple sclerosis (MS), with approximately 20% of MS patients experiencing ON as their initial clinical manifestation, and up to 50% of MS patients developing ON at some point during the course of the disease [1].The Optic Neuritis Treatment Trial (ONTT) demonstrated that, while the majority of ON patients experience visual recovery within several weeks of symptom onset, up to 40% of ON patients, however, may suffer some degree of permanent vision impairment [1,2,3]. The pathogenesis of ON involves a complex interaction of inflammatory and neurodegenerative processes. Histopathological studies have demonstrated that ON lesion are characterized by the infiltration of inflammatory cells, demyelination, and reactive gliosis in the acute phase, followed by chronic inflammation of the optic nerve axons with a relapsing and remitting course, eventually leading to axonal loss and neuronal damage in the chronic phase [4,5,6,7]. This neuronal damage, particularly to retinal ganglion cells (RGCs) and their axons, is thought to be the key element in the permanent vision loss observed in patients with ON [4]. 

There is considerable evidence supporting a possible role for the renin–angiotensin system (RAS), a key regulator of blood pressure and fluid balance, in the pathophysiology of inflammatory diseases of the central nervous system (CNS), including MS and ON [8]. The classical circulating RAS cascade involves the sequential cleavage of angiotensinogen to angiotensin I (Ang I) by renin, and Ang I is further transformed by angiotensin-converting enzymes (ACE) into different angiotensin cleavage products, such as Angiotensin II (Ang II) [8,9,10]. Ang II is the major effector molecule of the RAS and exerts its biological effects through Ang II type 1 and type 2 receptors (AT1R and AT2R) [8,9,10]. Ang(1-7) may counteract the effects of the activation of AT1R by Ang II via a G-protein coupled receptor, Mas (MasR) [8,11]. 

In addition to this systemic RAS, a local RAS has been identified in various tissues, including the brain, where it has been implicated in the modulation of neuroinflammation and neurodegeneration [8,11,12]. In the context of the brain RAS, Ang II has emerged as a key mediator of neuroinflammation, acting via AT1R to promote the activation and polarization of microglia towards a pro-inflammatory phenotype [13,14,15]. Microglia can shift between pro- and anti-inflammatory phenotypes according to environmental signals related to physiological or pathological conditions [12]. Therefore, Ang II can exacerbate neuroinflammation via the activation of the pro-inflammatory arm of the brain RAS [13,14,15]. However, this pro-inflammatory action of Ang II is counterbalanced by the Ang II/AT2R pathway and Ang(1-7)/MasR axis [13,14,15]. Dysregulation of the brain RAS, particularly an imbalance favoring the pro-inflammatory AT1R pathway, has been proposed to contribute to the pathogenesis of neurodegenerative and neuroinflammatory disorders of the CNS [12,16]. 

Prolylcarboxypeptidase (PRCP), also known as angiotensinogen C, is a serine protease that plays anti-hypertensive and anti-inflammatory roles via the inactivation of the RAS [17,18] PRCP hydrolyzes Ang II to Ang(1-7), thereby shifting the balance of the RAS towards the anti-inflammatory and neuroprotective Ang(1-7)/Mas pathway [17,18]. Notably, PRCP was identified as one of the astrocyte-secreted proteins in the glia secretome analysis (https://www.gliome.org, accessed on 7 April 2022), suggesting its potential role in the modulation of neuroinflammation in the CNS [19].

Given the involvement of the RAS in the pathophysiology of inflammatory diseases of the CNS and the anti-inflammatory role of PRCP within the RAS system, we hypothesized that PRCP may be upregulated in patients with ON as a compensatory mechanism to counteract neuroinflammation and mitigate neuronal damage. To test this hypothesis, we first investigated the expression of PRCP in the optic nerves of mice with experimental autoimmune optic neuritis (EAON), a well-established animal model of demyelinating ON. We then conducted a clinical study to measure the plasma level of PRCP and explored its relationship with visual outcomes and structural measurements of neuronal injury in RGCs in patients with idiopathic acute ON. To the best of our knowledge, this is the first study to investigate the role of PRCP in the context of demyelinating ON. Our findings provide novel insights into the potential involvement of PRCP in the pathophysiology of demyelinating ON and its ability as a biomarker of disease activity and prognosis. This study may inform the development of new therapeutic strategies targeting the RAS and PRCP pathway for the treatment of demyelinating ON and other inflammatory disorders of the CNS.

## 2. Materials and Methods

### 2.1. Animals—Experimental Autoimmune Optic Neuritis Model

Female C57BL/6 mice (7–8 weeks of age) were purchased from Samtaco (Osan, Republic of Korea). All animals were housed in groups of three per cage under specific pathogen-free conditions in a controlled environment (12 h light/dark cycle; 23 ± 2 °C; 50 ± 10% humidity) with free access to food and water. All animal experiments were conducted in compliance with the animal care guidelines of the National Institutes of Health and the ARRIVE guidelines, and were approved by the Institutional Animal Care and Use Committee of Kyungpook National University (approval number: KNU-2021-0001). EAON was induced as previously described [20]. Briefly, areas draining into the axillary and inguinal lymph nodes of the 7–8-week-old mice were injected subcutaneously with 200 µg of myelin oligodendrocyte glycoprotein (MOG35–55 peptide fragment MEVGWYRSPFSRVVHL YRNGK; GL Biochem Ltd., Shanghai, China) emulsified in a 100 µL solution containing 50% complete Freund’s adjuvant and 10 mg/mL of a heat-killed H37Ra strain of *Mycobacterium tuberculosis* (Difco Laboratories, Detroit, MI, USA) [20]. Pertussis toxin (List Biological Laboratories, Campbell, CA, USA) dissolved in PBS (200 ng/mouse) was injected intraperitoneally on the day of immunization and again at 48 h post-immunization [20]. The mice were examined for disease severity daily. The evaluation of disease severity was performed in a blinded fashion. Disease severity was scored using a 5-point scale as follows: grade 0 = no clinical signs; grade 1 = limp tail; grade 2 = weakness or incomplete paralysis of one or two hind limbs; grade 3 = complete hind limb paralysis; grade 4 = complete hind limb paralysis and weakness or paralysis of one or two front limbs; and grade 5 = moribund state or death [20]. The mice were sacrificed at day 14 post-immunization, corresponding to the peak of clinical disease, and their optic nerves were harvested for gene expression analysis. 

### 2.2. Real-Time PCR

The total RNA was extracted from the optic nerve tissue using TRIzol reagent (Invitrogen), in accordance with the manufacturer’s protocol. The RNA concentration and purity were assessed by spectrophotometry using a NanoDrop 2000 (Thermo Fisher Scientific, Waltham, MA, USA). The total RNA (0.5 µg) was reverse-transcribed into cDNA using Superscript II (Invitrogen) and oligo (dT) primers. Real-Time PCR was performed using the One Step SYBR PrimeScript RT-PCR Kit (Takara Bio Inc., Tokyo, Japan) with the ABI Prism 7500 Sequence Detection System (Applied Biosystems, Foster City, CA, USA). The specific primers were as follows; prcp forward: 5′-CCGCATTTGTCAGCCAGTC-3′, reverse: 5′-CCAAAGTGGTCAACCTT CTGTTC-3′. The PCR cycling conditions were as follows: 50 °C for 2 min, 95 °C for 2 min, followed by 40 cycles of 95 °C for 15 s and 60 °C for 1 min. The relative gene expression was calculated using the 2^−ΔΔCt^ method, with GAPDH as the reference gene and naive mice as the calibrator group.

### 2.3. Human Subjects

This retrospective study was approved by the Institutional Review Board of the Kyungpook National University Hospital (approval number: 2021-07-050) and adhered to the tenets of the Declaration of Helsinki. Informed consent was waived due to the retrospective nature of the study. 

We reviewed the medical records of patients diagnosed with acute demyelinating ON at the Department of Ophthalmology, Kyungpook National University Hospital, between September 2018 and August 2021. The inclusion criteria were: (1) first episode of unilateral ON; (2) age ≥ 18 years; and (3) follow-up period ≥ 6 months. The exclusion criteria were: (1) history of MS, neuromyelitis optica spectrum disorder (NMOSD), or other neuroinflammatory disorders; (2) positive serology for aquaporin-4 (AQP4) or myelin oligodendrocyte glycoprotein (MOG) antibodies; (3) presence of brain or spinal cord lesions consistent with MS or NMOSD on magnetic resonance imaging (MRI); (4) other ocular pathologies that could affect visual function; and (5) systemic comorbidities that could influence the RAS activity, such as hypertension, diabetes mellitus, renal disease, sarcoidosis, cardiovascular disease, and patients taking ACE inhibitors.

A total number of 34 patients consecutively diagnosed with a first attack of demyelinating ON were identified, of which 20 patients with idiopathic acute ON who met the eligibility criteria were included in this study. For each patient, we collected demographic data, clinical characteristics, and plasma samples at the time of ON diagnosis, before the initiation of treatment. ON was diagnosed based on the presence of clinical manifestations, such as an acute decrease in visual acuity, dyschromatopsia, or visual field defects consistent with optic neuropathy, with a relative afferent pupillary defect in the symptomatic eye [3]. Based on clinical, radiological, and antibody testing, the patients were diagnosed with idiopathic acute ON and received intravenous high-dose methylprednisolone (1 g/day) for 3 days with oral prednisolone (1 mg/kg) for 11 days according to the ONTT protocol [3]. 

Best-corrected visual acuity (BCVA) was measured using a Snellen chart and converted into logarithm of the minimum angle of resolution (logMAR) units for statistical analysis. The peripapillary retinal nerve fiber layer (RNFL) and macular ganglion cell layer plus inner plexiform layer (GCL+IPL) thicknesses were measured using spectral-domain optical coherence tomography (OCT) (Cirrus HD OCT, Carl Zeiss Meditec, Inc., Dublin, CA, USA) at diagnosis and 6 months after ON diagnosis. Differences in RNFL or macular GCL+IPL are defined as the thickness of the average RNFL or macular GCL+IPL measured at diagnosis minus the thickness of the average RNFL or macular GCL+IPL measured at 6 months after ON diagnosis. Twenty age- and sex-matched healthy controls with no history of ocular or neurological diseases were recruited from the hospital staff and their families. 

### 2.4. PRCP Measurement in Human Plasma by ELISA 

The PRCP levels were measured in plasma samples from the 20 patients with idiopathic acute ON and 20 healthy controls with Snellen visual acuities of 20/20. Blood samples of the patients were obtained on the day of ON diagnosis. Intravenous high-dose steroid therapy according to the ONTT regimen was initiated after the blood sample collection [3]. All patients had no previous neurological diseases or newly occurring neurologic symptoms related to their visual disturbance. 

The plasma PRCP levels were measured using a commercially available Sandwich ELISA kit (Cloud-clone Corp., Wuhan, China), according to the manufacturer’s instructions. The assays were run in 96-well plates using 100 μL of plasma (1:10 dilution). After incubation and washing steps, the biotinylated detection antibody, HRP conjugate, and TMB substrate were added sequentially. The reaction was stopped with the stop solution, and the optical density was read at 450 nm using a microplate reader (VersaMax, Molecular Devices, Sunnyvale, CA, USA). The PRCP concentrations (pg/mL) were calculated from the standard curve using a four-parameter logistic regression model. The assay’s detection range was 31.25–2000 pg/mL, with an intra-assay coefficient of variation (CV) of <8% and an inter-assay CV of <10%. All measurements were collected from duplicated assays. 

### 2.5. Statistical Analysis

Statistical analyses were performed using GraphPad Prism version 8.0 (GraphPad Software, La Jolla, CA, USA) and MedCalc version 19.8 (MedCalc Software Ltd., Ostend, Belgium). Continuous variables are presented as mean ± standard deviation (SD) or median and interquartile range (IQR), depending on the normality of the distribution. Categorical variables are expressed as frequencies and percentages. The Shapiro–Wilk test was used to assess the normality of the data. For the animal study, differences in the PRCP mRNA expression between naive and EAON mice were analyzed using the unpaired Student’s t-test (two-tailed). For the human study, comparisons of the plasma PRCP levels and other variables between the ON and control groups were performed using the Mann–Whitney U test. Correlations between plasma PRCP levels, LogMAR visual acuity, and OCT parameters such as RNFL and GCL+IPL thickness were assessed using Spearman’s rank correlation coefficient (ρ). To evaluate the prognostic value of plasma PRCP for ON recurrence, we constructed a receiver operating characteristic (ROC) curve and calculated the area under the curve (AUC) with 95% confidence intervals (CI). The optimal cut-off value was determined by maximizing the Youden index (sensitivity + specificity − 1). A *p*-value of <0.05 was considered to be statistically significant for all tests.

## 3. Results

### 3.1. Increased PRCP mRNA Expression in the Optic Nerves of EAON Mice 

**Figure 1:**
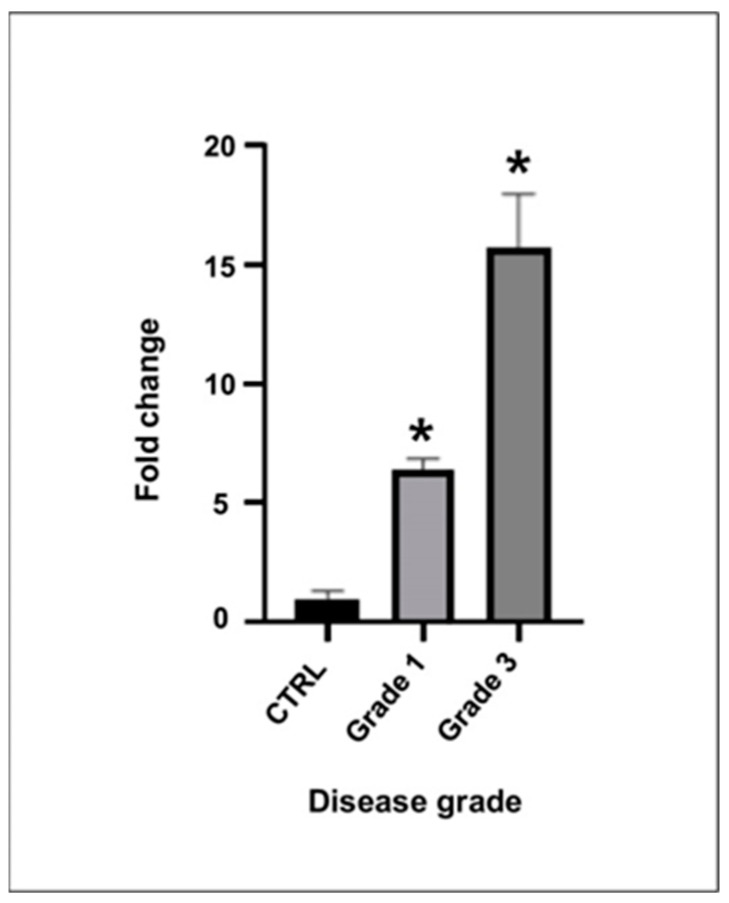
Upregulation of PRCP mRNA expression in the optic nerves of EAON-induced mice.

To investigate the potential involvement of PRCP in the pathogenesis of demyelinating ON, we first examined its mRNA expression in the optic nerves of mice with EAON, a well-established animal model that recapitulates the key features of human demyelinating ON [20]. The total RNA of the optic nerve tissue was collected when the mice showed typical grade 1 and 3 disease symptoms after immunization with MOG. A real-time PCR analysis revealed a significant upregulation of PRCP mRNA levels in the optic nerves of the EAON mice at the peak of clinical disease (day 14 post-immunization) compared to the naive controls (Figure 1). PRCP mRNA expression was significantly increased (*p* < 0.001) in the EAON mice, with clinical scores of 1 and 3, respectively, relative to the naive group. These results suggest that PRCP expression is induced in the optic nerve during the acute phase of EAON, and that its levels correlate with the severity of neurological deficits.

A real-time PCR analysis of the PRCP mRNA expression levels in the optic nerves of naive mice and EAON mice was performed, with clinical scores of 1 and 3 at day 14 post-immunization. Data are presented as fold changes relative to the naive group (set as 1) after normalization to GAPDH. Each data point represents an individual mouse (n = 6 per group). Horizontal lines indicate mean ± SD. One-way analysis of variance (ANOVA) was followed by Tukey post-hoc test. * *p* < 0.001 vs. naïve mice (CTRL). 

### 3.2. Increased Plasma PRCP Levels in Patients with Idiopathic Acute Optic Neuritis

Next, we sought to determine whether the upregulation of PRCP observed in the EAON model could be detected in the plasma of patients with acute idiopathic ON. We measured the plasma PRCP concentrations by ELISA in a cohort of 20 patients with idiopathic acute ON and 20 age- and sex-matched healthy controls. The demographic and clinical characteristics of the study participants are summarized in Table 1. There were no significant differences between the two groups in terms of age (*p* = 0.958) or sex distribution (*p* > 0.999). The mean BCVA at diagnosis was logMAR 1.03 in the ON group, indicating severe visual impairment due to ON attack. 

**Figure 2:**
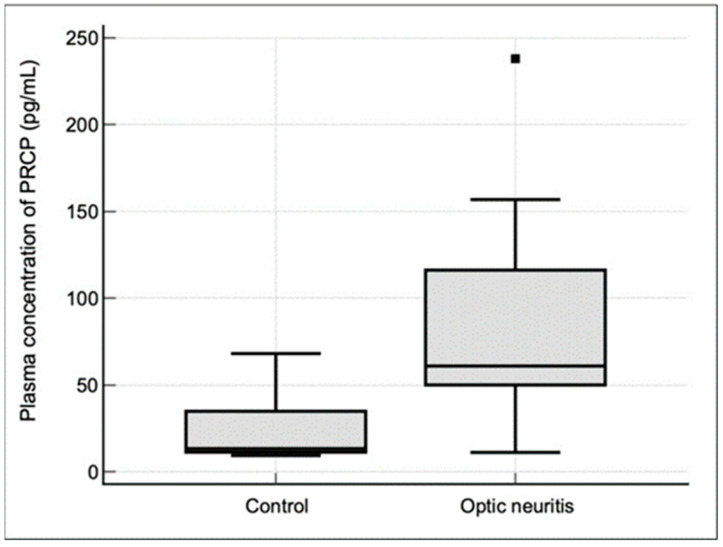
Increased plasma PRCP levels in patients with idiopathic acute ON.

As shown in Figure 2, patients with idiopathic acute ON had significantly higher plasma PRCP levels compared to the healthy controls (median [IQR]: 83.80 [62.21–112.80] vs. 22.80 [18.30–28.65] pg/mL; *p* < 0.0001). The median PRCP concentration in the ON group was 3.7-fold higher than that in the control group, consistent with the magnitude of PRCP upregulation observed in the optic nerves of the EAON mice. These findings indicate that PRCP is systemically elevated in the acute phase of ON, and that its plasma levels may serve as a biomarker of disease activity.

**Table 1:** Demographics and clinical characteristics of the study participants.

	Patients with ON	Controls
Number	20	20
Sex (male: female)	7:13	7:13
Age (years, mean ± SD)	42.0 ± 17.4	41.8 ± 17.4
Mean BCVA at diagnosis (LogMAR, mean ± SD)	1.03 ± 0.76	NA
Mean BCVA at 6 months after diagnosis (LogMAR, mean ± SD)	0.42 ± 0.63	NA
Recurrence (number, %)	4 (20%)	NA

*p*-values were calculated using the Mann–Whitney U test for continuous variables and Fisher’s exact test for categorical variables. ON, optic neuritis; BCVA, best-corrected visual acuity; logMAR, logarithm of the minimum angle of resolution; and NA, not applicable.

Measurements of plasma PRCP levels by ELISA in healthy controls (n = 20) and patients with idiopathic acute ON (n = 20) were performed. The median values were significantly different between the two groups (22.80 vs. 83.80 pg/mL). The Mann–Whitney U test was used (*p* < 0.0001.)

### 3.3. Plasma PRCP Levels Correlate with Visual Outcomes and Structural Measurements of Neuronal Damage

**Figure 3:**
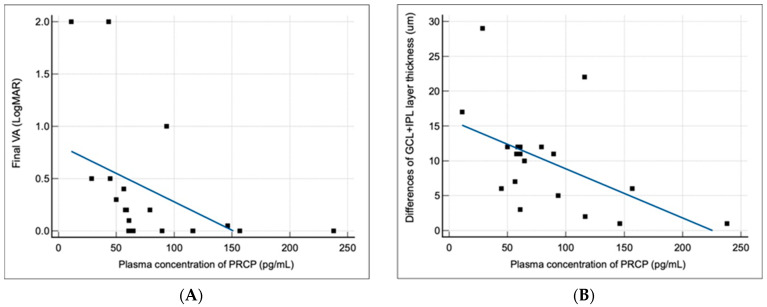
(**A**). Patients with higher plasma PRCP levels demonstrated better visual outcomes at 6 months after diagnosis. (**B**). Patients with higher plasma PRCP levels demonstrated less loss of GCL+IPL layer thickness during ON attack.

To investigate the clinical relevance of plasma PRCP levels in idiopathic acute ON, we examined their relationship with visual outcomes and structural measurements of neuronal damage at 6 months after the acute episode of ON. Spearman correlation analysis revealed a significant negative correlation between baseline plasma PRCP levels and BCVA (logMAR acuity) at 6 months (ρ = −0.51, *p* = 0.022), indicating that higher PRCP levels at presentation were associated with better visual outcomes (Figure 3A). However, there was no correlation between plasma PRCP levels and visual acuities at diagnosis (LogMAR) (*p* > 0.05). 

Similarly, there was a significant negative correlation between plasma PRCP levels and changes in GCL+IPL thickness from baseline to 6 months (ρ = −0.60, *p* = 0.005), suggesting that higher PRCP levels were associated with less RGC layer loss (Figure 3B). In contrast, there was no significant correlation between plasma PRCP levels and changes in RNFL thickness (ρ = −0.34, *p* = 0.143), which may reflect the confounding effect of axonal swelling in the acute phase of ON.

Scatterplots were constructed showing the relationship between baseline plasma PRCP levels and (3A) BCVA (logMAR acuity) at 6 months and (3B) changes in GCL+IPL thickness from baseline to 6 months in patients with idiopathic acute ON (n = 20). Spearman’s rank correlation coefficient (ρ) and p-values are indicated. GCL, ganglion cell layer; IPL, inner plexiform layer; differences of GCL+IPL thickness = GCL+IPL thickness at diagnosis minus GCL+IPL thickness at final visit; and ON, optic neuritis

### 3.4. Plasma PRCP Levels Predict the Risk of ON Recurrence

**Figure 4:**
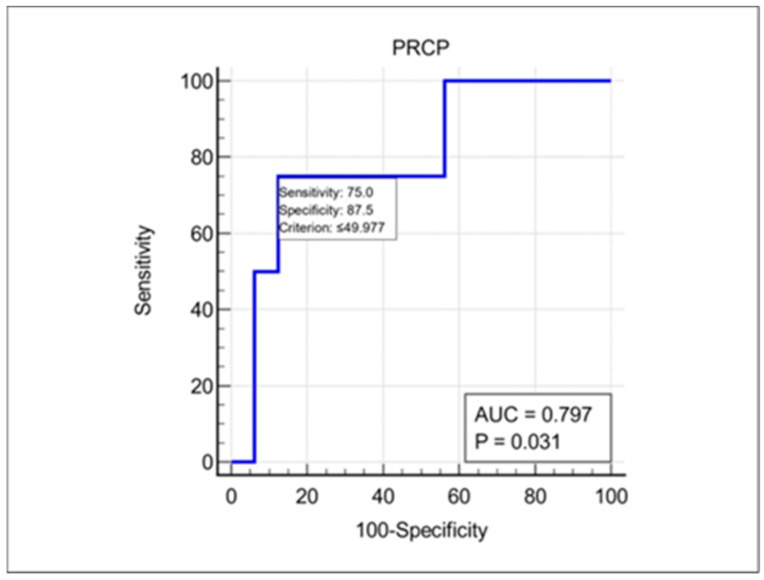
Prognostic value of plasma PRCP levels for the recurrence of ON.

During the follow-up period (median [IQR]: 18 (12–24) months), 4 out of 20 patients (20%) experienced at least one recurrent episode of ON in the same or contralateral eye. To assess the prognostic value of plasma PRCP levels for ON recurrence, we performed an ROC curve analysis. As shown in Figure 4, the area under the ROC curve (AUC) was 0.797 (95% CI: 0.560–0.941, *p* = 0.031), indicating a good discriminatory ability. The optimal cut-off value of plasma PRCP for predicting ON recurrence was 49.98 pg/mL, with a sensitivity of 100% and a specificity of 75%. Notably, all 4 patients who developed recurrent ON had baseline plasma PRCP levels below this cut-off value, while 12 out of 16 patients (75%) who remained recurrence-free had PRCP levels above the cut-off. These findings suggest that low plasma PRCP levels at presentation may be a risk factor for ON recurrence, and that PRCP may be useful as a prognostic biomarker in this setting.

An ROC curve analysis of baseline plasma PRCP levels for predicting the risk of ON recurrence within the follow-up period was performed. The optimal cut-off value of 49.98 pg/mL was determined by maximizing the Youden index. AUC, area under the curve; CI, confidence interval.

## 4. Discussion

In this study, we investigated the expression and clinical significance of PRCP, a key enzyme involved in the metabolism of Ang II in the context of demyelinating ON. Our main findings were as follows. (1) PRCP mRNA expression was significantly upregulated in the optic nerves of EAON-induced mice compared to that of naïve mice, and was correlated with the severity of neurological deficits; (2) plasma PRCP concentrations were significantly elevated in patients with acute idiopathic ON compared to healthy controls; and (3) higher plasma PRCP levels at presentation were associated with better visual outcomes, less RGC layer loss, and a lower risk of recurrence in patients with idiopathic acute ON. To our knowledge, this is the first study to implicate PRCP in the pathophysiology of demyelinating ON and to suggest its potential utility as a biomarker of disease activity and prognosis.

Our findings in the EAON model are consistent with previous studies demonstrating the involvement of the RAS in the pathogenesis of ON and other neuroinflammatory disorders [8,9,10]. In particular, our observation of increased PRCP mRNA expression in the optic nerves in EAON mice parallels the previously reported upregulation of Ang II and its receptors in EAON-induced mice [8]. This study showed that the administration of candesartan, an Ang II receptor antagonist, markedly attenuated the infiltration of inflammatory cells and demyelination in the optic nerves of EAON-induced mice [8]. Additionally, they reported that candesartan treatment significantly reduced neuronal loss in the RGC layer and improved visual function in EAON-induced mice [8]. Thus, they suggested a contribution of the pro-inflammatory action of Ang II to the development of ON and the inhibition of neuroinflammation in the CNS by the administration of candesartan, an Ang II receptor antagonist [8]. These results imply that inhibition of the RAS might be effective in decreasing the incidence and severity of neuroinflammatory disease in the CNS [8]. Given the known role of PRCP in converting Ang II into Ang(1-7), which has anti-inflammatory and neuroprotective effects mediated by the Mas receptor, it is tempting to speculate that the induction of PRCP in EAON represents a compensatory mechanism to counteract the pro-inflammatory actions of angiotensin II [17,18]. This hypothesis is supported by our finding that PRCP mRNA levels correlated positively with the severity of neurological deficits in EAON-induced mice, suggesting that PRCP upregulation may be a marker of disease activity.

The translational relevance of our findings in the EAON model is underscored by the observation of elevated plasma PRCP levels in patients with acute idiopathic ON. The magnitude of PRCP upregulation in the ON patients compared to the healthy controls (3.7-fold) was similar to that observed in the optic nerves of the EAON mice, suggesting that PRCP induction is a conserved feature of ON pathogenesis across species. Moreover, the fact that the plasma PRCP levels were increased in the ON patients indicates that the local upregulation of PRCP in the optic nerve is accompanied by a systemic response that can be detected in the peripheral circulation. This finding raises the possibility that plasma PRCP could serve as an accessible biomarker in idiopathic acute ON, which is currently diagnosed based on clinical criteria and imaging studies that may not always be conclusive [1,2,3]. However, the exact mechanism responsible for the increase in plasma PRCP levels in patients with idiopathic acute ON remains elusive.

The clinical significance of plasma PRCP levels in idiopathic acute ON is supported by our finding that higher PRCP levels were associated with better visual outcomes and less RGC layer loss at 6 months after an episode of ON. This observation suggests that PRCP may have a neuroprotective effect, possibly by counteracting the pro-inflammatory effects of Ang II and shifting the balance of the RAS towards the anti-inflammatory effect of Ang(1-7) [17,18]. Our results correspond with those of Ghanbari et al., who reported that the serum level of the ACE was significantly higher in patients with idiopathic acute ON than in normal controls [21]. They also showed that the serum ACE in patients with ON was notably higher in patients with better visual acuity; however, serum ACE level did not vary with age, sex, laterality of the affected eye, and disc swelling [21]. Their results highlighted the possible roles of ACE activity and the RAS in the pathogenesis of idiopathic acute ON. Alternatively, higher PRCP levels may reflect a more robust compensatory response to neuroinflammation, which could limit the extent of tissue damage and promote recovery. The lack of correlation between plasma PRCP levels and changes in peripapillary RNFL thickness may be due to the confounding effect of axonal swelling in the acute phase of ON, which can mask the extent of axonal loss in the affected eye. In contrast, the macular GCL+IPL thickness is a more reliable measure of neuronal damage in patients with ON, as it reflects the integrity of the RGC bodies and dendrites, which are not affected by edema. 

Another novel finding of our study was that plasma PRCP levels at presentation may predict the risk of ON recurrence. Using an ROC curve analysis, we identified a cut-off value of 49.98 pg/mL that discriminated between patients who did and did not experience a recurrent episode of ON during the follow-up period, with a high sensitivity and moderate specificity. This finding suggests that low plasma PRCP levels may be a risk factor for ON recurrence, possibly reflecting an inadequate compensatory response to neuroinflammation that leads to persistent tissue damage and increased susceptibility to future relapses of ON. If validated in larger cohorts, plasma PRCP could be a useful prognostic biomarker, helping to identify patients who may benefit from more aggressive immunotherapy or closer monitoring in patients with idiopathic acute ON. It is well-known that MOG-IgG positivity is related to the recurrence of ON [22]. However, there is no known diagnostic autoantibody, such as AQP4 or MOG-IgG, in patients with idiopathic acute ON. Therefore, measuring plasma PRCP levels may be beneficial in predicting a risk of ON recurrence in patients with idiopathic acute ON.

The strengths of our study include the use of a well-characterized animal model of demyelinating ON to investigate the local expression of PRCP in the optic nerve, the inclusion of age- and sex-matched healthy controls to establish the normal range of plasma PRCP levels, and the longitudinal assessment of visual outcomes and structural measures of neuronal damage in idiopathic acute ON patients. However, our study also has several limitations that should be acknowledged. First, the sample size of the human study was relatively small, which may limit the generalizability of our findings and the power to detect associations between plasma PRCP levels and clinical outcomes of idiopathic acute ON. Second, the follow-up period was relatively short (median 18 months), which may not capture the full spectrum of long-term outcomes in ON patients. Third, we did not investigate the expression of other components of the RAS, such as Ang II and its receptors, which could provide further insights into the role of the RAS system in the pathogenesis of ON. Finally, we did not assess the relationship between plasma PRCP levels and other biomarkers of neuroinflammation and neurodegeneration. Future studies should aim to replicate our findings in larger and more diverse cohorts of ON patients, and to investigate the mechanisms underlying the upregulation and neuroprotective effects of PRCP in this context. Additionally, longitudinal studies are needed to evaluate the long-term prognostic value of plasma PRCP levels in idiopathic acute ON, and to assess their relationship with other biomarkers and clinical outcomes.

In conclusion, our study provides the first evidence implicating PRCP in the pathophysiology of demyelinating ON and suggests that plasma PRCP levels may be a useful biomarker of disease activity and prognosis in idiopathic acute ON. The current study demonstrated that the plasma PRCP levels in patients with idiopathic acute ON were significantly increased compared to those in healthy controls. Higher plasma PRCP levels at presentation were associated with better visual outcomes, less RGC layer loss, and a lower risk of recurrence in patients with idiopathic acute ON. Our findings support the notion that the RAS plays a role in the pathogenesis of demyelinating ON, and that targeting the RAS system may be a promising therapeutic strategy for neuroinflammatory diseases of the CNS, including ON. Considering these results, plasma PRCP levels may be a potentially valuable biomarker for predicting final visual outcomes and risks of recurrence in patients with idiopathic acute ON. Finally, the therapeutic potential of targeting PRCP or other components of the RAS in ON should be explored in preclinical and clinical studies.

## Data Availability

The datasets generated and/or analyzed during the current study are available in the OSF repository, DOI 10.17605/OSF.IO/FE3HR (https://osf.io/fe3hr/, accessed on 8 June 2022).
